# Annual Address of the President of the American Medical Association

**Published:** 1856-08

**Authors:** Geo. B. Wood

**Affiliations:** Prof., &c., &c., of Philadelphia


### SELECTIONS.

*Annual Address of the President of the American Medical Association.* By Geo. B. Wood, M.D., Prof. &c., &c., of Philadelphia.

Custom demands, as one of the expiring duties of your presiding officer, that he should leave a legacy at least of good wishes, if not of something more valuable behind him. In compliance with this duty, I propose to say a few parting words, which, whatever else they may convey to you, will assuredly not interpret duly the sentiments of him who utters them, unless they make you sensible of his grateful and most kindly feelings towards his fellow-members, and of his zealous interest in the great objects of our Association.

The present is a suitable occasion for taking a survey of the Association; for looking around towards the boundaries of its labors, interests, and duties, and noting whether something may not present itself in the view, which may profitably occupy, for a few minutes, our serious and earnest attention. Let us first throw a comparative glance from the present backward to the past. Perhaps by so doing we may be better prepared to look forward intelligently into the future.

Have the hopes with which the Association set out in its mission of self-imposed duty been fulfilled? Has the loud call which it sent forth through the nation, startling the profession from its uneasy slumber, succeeded in awakening it thoroughly to a sense of its high responsibilities, and arousing a determined spirit of progress? Or has it died away in gradually-diminishing echoes, leaving but a drowsy memory of that spirit-stirring appeal? Have the annual gatherings of the elect of the profession, their joint deliberations in council, their various legislation, the practical inquiry set on foot or encouraged, not omitting their exploits at the festal board, and kindly interchange of thought and sentiment in social assemblage; have all these been without fruit? Have they been the mere course of a phantom ship through the ocean of human events, leaving no track in its passage, and bearing no freight onward to its destination?

Were we to listen to the clamors of opposition, the whisperings of discontent, or the murmured disappointent of an over-excited expectation, we might be disposed to give to these questions an unfavorable answer; to cease our struggles for an unattainable good; and with the wings of the spirit folded, and its head drooping, to submit in sadness to an inexorable destiny, chaining us in submission to all present evils, and jealous even of a glance towards the higher and the better.

But, happily, such is not the voice of a clear and unbiassed judgment. It is true that the Association has not accomplished the whole of what it aimed at. Like all young things, conscious of a stirring life within, and feeling no limits to its yet untried powers, it hoped and strove beyond the possible; it struck in its soaring flight against the iron wall of circumstance, and for a time, at least, fell back, stunned though not crushed, into humbler aims. Yet, even as regards medical education, which is the main point of failure, its efforts have not been all thrown away. Some advance, however small, has, I think, been already made; and bread, moreover, has been cast upon the waters to be found after many days.

But outside of this vexed subject, much, very much, has been accomplished. I will not appeal to the ponderous volumes of our Transactions. They speak for themselves. To say that there is no chaff among their solid contents, would be to say what is neither now nor ever has been true of any large book, with one solitary exception. But I believe that all present will join me in the opinion, that one who searches these records with a sincere and candid spirit will find in them much that is good; much that may warrant the self-congratulation of the Association for having originated, or called it forth.

But, whatever credit may be given to these living witnesses of our labors, one fact is evident, that the medical mind has been aroused; that the spirit of improvement has breathed upon the masses of the profession, and everywhere scattered germs, which are now developing, and will probably hereafter continue to develop, even in a still higher ratio, into earnest efforts for self-culture and general advancement.

Stagnation, in the moral as in the physical world, generates corruption. Agitation, though often in its extremes a cause of evil, and sometimes of unspeakable present wretchedness, generally purifies in the end, and, if restrained within due limits, is a source of unmixed good. The medical mind, anterior to the birth of this Association, was in a state of comparative inertia. In all the departments of the profession, the educational as well as the practical, material interests began to predominate. There was danger that the profession might sink to the level of a mere business. Noble aims; high aspirations; the general good; the spirit of self-sacrifice; these began to be looked on as wordy inflations. The great struggle seemed to be, in the teaching department, to gather pupils; in the practical, to gather patients: in both, to swell the pockets. Stagnation of the professional spirit was breeding noxious influence in its motionless depths. No wonder that quackery loomed upward as regular medicine began to sink. There was danger that the public might be able to see little difference between them; and the fact is, that the line of demarkation was not very distinct, even to the professional eye. They ran into each other, at their extremes, by quite insensible shades.

But the Association arose, and a new spirit was awakened. Many had been watching this apparent abasement of the profession with sorrow; but they were powerless in their isolation. No sooner had the flag of the Association been given to the breeze, than they hastened to join its standard. From all quarters, and from the remotest bounds of the country, volunteers poured in to join this great crusade against the evils which had been usurping the sacred places of the profession. The mass of medical society was moved to its very depths. Hundreds upon hundreds came forth from their sheltering privacy, and threw their souls into the grand movement which was to reconquer, to purify, and regenerate the prostrated glory of their calling. The feeble voice of opposition was heard for a moment; but was soon drowned in the overwhelming shouts of the masses, crying out, Onward! Onward! Even the advocates of the material principle, who could not raise their souls above the level of dollars and cents, found it expedient to chime in for a time with the almost universal voice; and to the enthusiastic it seemed as though a professional millenium was approaching. I need not follow the march of the crusade. I need not recal the varied experience which has but confirmed that of all other revolutionary uprisings, that, except under the influence of a power higher than human, which can regenerate the hearts of men, whatever temporary change may be made in the surface of things, in mere form and arrangement, it is only by the slow working of time that radical and lasting reforms can be effected. Who ever beheld a great nation made by a written constitution? We have had paper republics as thick as the leaves in Vallombrosa; but where, and what are they now? To make a great and free nation, the people must have the principles of greatness and freedom implanted in their hearts. So is it with lesser associations. It is vain to alter forms, unless the substance is altered too. The Association has discovered this truth. It no longer seeks to work miracles, but is content with following the methods of nature and providence. It has done a great thing in beginning the movement. It is doing what it can to further that movement, and to consolidate its results.

Who is there that has lived and observed through the last ten or fifteen years, who cannot see that our profession has been moving onward and upward since its great awakening; perhaps slowly, perhaps now and then halting, but on the whole advancing, and with an irresistible force, because it is that of the mass. It is not now a few leaders who are kindling by their own enthusiasm a feeble and temporary blaze of excitement in the multitude; dragging them forward as with cords by their own strong zeal and fiery spirit; it is the inborn soul which is animating the great body, and carrying it forward in its legitimate course.

Had the Association done nothing else, I will not say than originating, but even than aiding and concentrating this rising up of the profession, it would have performed a service entitling it to everlasting gratitude, and to an imperishable name in the medical annals of our country.

A great benefit conferred on the profession by the Association, was the preparation and adoption of a code of medical ethics. I need not say to *you*, that this code is merely an expression of the great principles of truth, justice, and honor, in their application to the relations of physicians to one another, their patients, and the public. It is the voice of wisdom and experience speaking from the past, and meets a ready response in the breast of every man possessed of a good heart, a sound judgment, and correct moral principle. Should any one find a repugnance to the observance of its rules rising up within him, let him for a moment reflect, whether this may not spring from some evil source in himself; whether it may not be the result rather of an unwillingness to make what he may deem a sacrifice at their suggestion, than of a real conviction of their injustice or impropriety. Which is more likely to be true; the unbiassed and unselfish judgment of the wisest and most experienced in the profession, or an individual decision which may at least be suspected of a selfish basis, and of which no man, if his interests or his feelings are in any degree involved, can say that it is quite pure; for no man can judge impartially in his own case. A becoming modesty would lead him to suspect that the fault might be in himself, and, a becoming spirit, to search into the depths of his own heart for the root of evil, and to pluck it out if discovered. I have no doubt that a full observance of these rules would tend more than any one thing else to maintain harmony in the profession and to elevate it in the public esteem. It would render impossible those unseemly disputes, founded on petty jealousies and supposed opposition of interests, which, probably beyond any other single cause, expose the profession to obloquy and ridicule. A copy of the Code should be placed in the hands of every young man about to enter upon the practice of medicine, with the urgent advice that he should make it the guide of his professional life; that he should not only regulate his conduct in conformity with its precepts, but should educate his heart into a real preference for them. Would it not be an object worthy of the attention of the Association to provide for such a distribution; at least by the publication of a large edition of the Code, to put it in the power of individuals or societies, who might be disposed to engage in this work of beneficence, to do so with as little cost to themselves as possible? I do honestly believe, that, to a young physician, going forth into a life full of moral conflicts, the wearing of this ægis would be one of his surest defences; that, next to the holy Scriptures and the grace of God, it would serve most effectually to guard him from evil.

Not one of the least advantages of the Association is, that, representing as it may be said to do the medical profession of the country, its voice, when nearly or quite unanimous, will be considered as that of the whole medical body, and thus have weight both in the community at large, and in the legislative councils of the nation. It is only thus that the profession can make their special opinions and wishes known and felt. I have been told that the representations of the Association had much weight in determining a satisfactory arrangement of the question respecting the relative rank of the surgeons in the navy. It is to be presumed that the patriotic physician, who brought before Congress the memorable measure for establishing a general inspection of imported drugs, was materially aided in carrying it through by the approving voice of the profession speaking in the memorial from this body. On another occasion you were heard, through your resolutions, pleading in the halls of Congress in favor of a great measure of honesty and justice, when you petitioned for an international copyright law between the United States and Great Britain; and, should such a law ever be passed, it will not be claiming too much for the Association to say that it will have contributed to that result. Your resolutions, from time to time, in advocacy of a system of registration of births, deaths, &c., have probably also added something to the mass of influence which has brought legislation to bear on this most important subject, though, it must be acknowledged, hitherto but very partially, and, with some honorable exceptions, ineffectually.

There is one other view of the beneficial influence of our great gatherings which I cannot pass unnoticed.

The effect of isolation is well-known in breeding excessive self-respect, distrust of others, and narrow, selfish, and sectional views and feelings. Man is naturally gregarious; and it is only in association that his nature can receive its full development; that the seeds of the better qualities within him can be made to germinate, and the qualities themselves to grow up, under culture, into their just magnitude and proportions.

Our Association brings together many who would otherwise never meet, from sections remote from each other, and differing much in views, habits, and feelings. We come, partly at least, for relaxation from the cares and toils of business, prepared and desirous to be pleased. Each one naturally, and without design, turns out the fairest side of his character, "his silver lining to the sun;" and all, consequently, make and receive favorable and kindly impressions. Each place selected for our meetings feels its character for hospitality involved in the reception of its guests, and every effort is made to extend all proper courtesies and kindnesses to the assembled representatives of the profession. In parting, therefore, we carry with us friendly remembrances of one another, and of the place of assemblage, to our several far-separated homes. These remembrances serve as so many cords not only to bind the members of the profession together in one harmonious whole, but also, intertwined with other similar agencies, to counteract the centrifugal tendencies of our political system, and to keep it moving onward, each part in its due place, in that majestic course which, while shedding beneficent influences throughout its own great circle, attracts the admiring and hopeful gaze of humanity everywhere.

Having thus hastily scanned the present and past of the Association let us turn our thoughts briefly towards the future. A few words will convey all that I have to address to your attention.

It seems to me that experience should have taught us this one lesson; not to aim at once at sweeping changes, but, having determined what great objects are desirable, to keep these always in view, and, by the persevering use of such influences as may be at our command, securing one point in advance, before hastening to another, to move on slowly but steadily to our ends. These must ever be the improvement of the profession itself, the advancement of medical science, and the promotion of the public good, so far as that may, in any degree, be connected with our special pursuit. Each of these three points requires a brief notice.

In the improvement of the profession, the Association has from its foundation recognized, as an essential element of success, a higher degree of qualification in those who are to become its members. But for the attainment of this object they can use no coercive measures. The only power they can exercise is that of opinion. Our only appeal is to the judgment and conscience of those concerned. But much may in time be done in this way. It is impossible that intelligent and honorable individuals, possessed of that share of conscientiousness which belongs to most men, and is certainly not deficient in our profession, should long resist such appeals, proceeding from a source so worthy of respeet as this. Let us reiterate, from time to time, our convictions of the necessity for improved preparatory education, for a longer devotion to the proper studies of the profession, for a junction of clinical with a didactic instruction, and finally for something more than a mere nominal examination before admission to the honor of the doctorate, or the privileges of a license to practice; points which have ever been insisted on by the association; let us, I say, reiterate these convictions; and like slowly dropping water, they will at length, however gradually, wear their way through the hardest incrustation of prejudice, interest, indolence, or indifference, and reach the conscience with irresistible effect. While bringing to bear upon this resistance, the considerations of reason, duty, honor, and even an enlightened self-interest, we must carefully avoid all violence of procedure, as likely only to add the hostility of passion to other opposing influences. By this course universal opinion will be gradually conciliated; and interest itself will find its own ends best promoted by compliance with the general will. Already some advance has been gained in this direction; and the Association, by perseverance, may yet see all reasonable wishes accomplished.

In relation to other measures for elevating the character and increasing the efficiency of the profession, there appears to me nothing more at present for the Association to do, than to go on as it has begun. Its continued existence alone is a great good; for it is annually bringing large numbers, simply through membership in its body, to participate in its feelings, and to acknowledge its obligations. Let us then maintain unshrinkingly the standard of professional honor and morals that we have erected, and decline association with those who will not recognize that standard, or having recognized, abandon it. Let us adhere unswervingly to the line which has been drawn between regular and irregular medicine, and treat the practitioners of the latter with the silent disregard they merit. This is the only course for the regular practitioner. To wage a war of words with quackery, is to do what it most delights in. It would be to contend, under the government of honor and principle, with antagonists who acknowledge no such restraints. In our private intercourse with friends and patients, we may explain the grounds of difference between ourselves and the irregulars, may demonstrate the absurdity of their pretensions, the danger of their practice, and the iniquity of their conduct; in short, may endeavor to enlighten whatever light is acceptable, or can penetrate. We may even, if the public interest seem to require it, put forth refutations of false doctrine and assertion, and expoposure of subterfuge, trickery, and imposture; but with the irregulars themselves we should enter into no relation, whether of friendship or hostility. I do not say that there may not be honorable and honest, though ignorant or bewildered, men among them. But we cannot discriminate. With the presumed advantages of their association, they must be content to take also the disgrace.

There is a point to which I would call the attention of the members of the Association individually. We have been called *Allopathists,* in contradistinction to a sect of irregular practitioners who have taken to themselves the title of *Homœopathists;* the latter term signifying that its professors treat disease by influences similar in their effects to the disease itself; the former that *other*, and of course dissimilar influences are used. It must be remembered, that the designation was not adopted by ourselves, but conferred upon us by Hahnemann and his followers. The intention was obvious. It was to place the regular profession, and their own scheme, upon a similar basis. They practised on one principle, we on a different and somewhat opposite principle. They graciously allowed that our principle was not altogether ineffective; that we did sometimes cure our patients; but theirs was sounder in theory and more successful in practice. Now, by recognizing the name, we necessarily recognize the principle also, and thus put ourselves in a false position. In deciding between them and us, the ignorant masses think they are deciding between two systems, neither of which they understand, but of which they must judge, upon the grounds of relative success. Diseases often get well of themselves, if left alone. The genuine homœopathist leaves them alone, and they often consequently terminate in recovery. This success is magnified by methods well understood; and multitudes are thus led astray, especially among the delicate and refined, who abominate the taste of medicine themselves, and are equally averse to the task of forcing it down the reluctant throats of their children. But we are *not* allopathists. The regular practice of medicine is based on no such dogma, and no exclusive dogma whatever. We profess to be intelligent men, who seek knowledge, in reference to the cure of disease, wherever we can find it, and, in our search, are bound by no other limits than those of truth and honor. We should not hesitate to receive it from the homœopathists, had they any to offer. We would pick it up from the filthiest common-sewer of quackery; for, like the diamond, it has this excellent quality, that no surrounding filth defiles it, and it comes out pure and sparkling, even from the kennel. This is the light in which the medical profession should present itself to the community. We are men who have sought in every possible way to qualify ourselves for the care of their health. We present them, in our diplomas, the evidence that we have gained sufficient knowledge to be trusted with this great charge; and we stand pledged before them to extend our knowledge and increase our skill, as far as may lie in our power. Membership in our honorable profession is the proof we offer, that we are no false pretenders, no interested deceivers; but upright men, intent on the performance of our professional duties. This the people can understand. But when we designate ourselves as *allopathists*, they may well ask, in what, are you better than any other medical sect, than the *homœopathists*, the *hydropathists*, the *Thomsonians*, the *eclectics?* Let us discard, therefore, the false epithet. Let us not only never employ it ourselves, but show that, when applied to us by others, it is inappropriate and offensive, and that the use of it in future would be contrary to gentlemanly courtesy, and the proprieties of cultivated society. I say again, we are not *allopathists;* we are simply *regular practitioners of medicine*, claiming to be honest and honorable—in other words, to be gentlemen.

The efficiency of our profession is to be increased not only by increasing its qualifications, but also by all upright measures calculated to win the public confidence, and thus widen the field of our operations. In this respect, I do not know that the Association can do better than to persevere as it has begun; and, by the propriety and dignity with which it conducts its own proceedings, to show to the world the high influences under which the profession acts, and domonstrate that it possesses those qualities of self-government, so useful to the medical practitioner, and so characteristic of the gentleman in all his relations.

The improvement of the *science* of medicine, has always been a favorite object of the Association. The appointment of committees to investigate and report on certain stated subjects, the reception of voluntary communications, the offering of prizes to competing contributors, and the publication of our Transactions annually, are the means employed for this purpose, and I have nothing better to suggest.

The remaining point for consideration, is the promotion of the public good. Happily, such is the nature of our profession, that the more we improve ourselves, the better do we fulfil this great duty. But there is something else to be done. There are certain great interests of the community, relating to their health, of which medical men are the only good judges, and the various influences affecting which, they only can duly appreciate. Upon these points it is our duty to be ever on the watch, and not only like faithful sentinels, to give notice of danger, but, like heaven-appointed agents, as we are, to use our best efforts and influence to prevent or remove it, and, in every practicable way, to guard the public health.

To the establishment of a general system of registration throughout the country, our attention has already been given. We should not relax our efforts, until the great end has been accomplished.

There is another subject deserving of our most serious consideration. You are all aware what advances have recently been made by the small-pox in many parts of our country. Thousands are perishing annually, for whose deaths we are, as a profession, in some degree accountable. There is no occasion for this mortality. Vaccination and revaccination, duly performed, and under proper circumstances, are, I will not say an absolutely certain, but a very nearly certain, safeguard. I have never known of death from small-pox, after an efficient revaccination; and only one instance of the occurrence of varioloid. But the profession and the community have both been too careless upon this point. Food for the pestilence has been allowed to accumulate; and it has been rioting with fearful results in many parts of our country. The profession should rouse itself from this apathy, and warn the community everywhere of the danger, while offering them the means of security. We may be accused of self-interest in urging this measure of precaution; as our own instrumentality may be necessary, and must be compensated where the means exist. But a moment's reflection must convince the most stupid, that it would be much more to our pecuniary interest to attend a protracted case of small-pox, than to perform a trifling operation, which is to prevent it. There are, however, many occasions, in which it is necessary to do our duty at the risk of obloquy; and this is one.

But, perhaps, I have been somewhat unjust to the profession.

The people have, in many places, and probably in some degree in almost all, chosen other guardians of their health and rejected our offered aid. It has happened to me to become acquainted with one neighborhood in which small-pox has recently prevailed; but not a single case occurred within the circuit of the regular physician's practice. Those families only suffered who had entrusted the care of their health to an empiric, who, for aught I know, may have been ignorant alike of small-pox and of vaccination. It is highly probable that many of those who now hear me could give a similar account of their own neighborhoods. The public should take this subject into their hands. Provision should be made, with legislative sanction, for universal vaccination. If the evil were confined exclusively to the negligent individual, the public might possibly have no right to interfere. But whole communities suffer, and Government may and ought to step in for their protection. A man is prohibited by law from setting fire to his own house, because a neighbor's may suffer. Which is the greater evil, that our house should burn, or our families perish with small-pox? It might be impossible in this country to establish a system of compulsory vaccination; but legislation might go far towards attaining the same end without this obnoxious feature. Time, however, does not permit me to follow this interesting subject in all its ramifications. I must content myself with having introduced it to your notice. If the profession can do nothing more, they can at least raise a warning voice everywhere; and this will be doing much.

I must close with begging you to excuse the length into which I have been drawn in the discussion of the important points that have engaged our attention. I intended to be very brief; but few men, when they have taken their pen in hand, can say to the flowing tide of their thoughts, "thus far shalt thou go, and no further." Allow me, in a few parting words, to thank you warmly for your attention, and to express the hope that our labors, during the present session, may tend to confirm the good that has been done, and to carry us still further onward in the great road of progress; so that, hereafter, the meeting at Detroit may be remembered as one, at which we may all be gratified and proud to have assisted.

